# Exploring the burden of fatal drowning and data characteristics in three high income countries: Australia, Canada and New Zealand

**DOI:** 10.1186/s12889-019-7152-z

**Published:** 2019-06-21

**Authors:** Amy E. Peden, Richard C. Franklin, Tessa Clemens

**Affiliations:** 1 Royal Life Saving Society – Australia, PO Box 558, Broadway, New South Wales Australia; 2 College of Public Health, Medical and Veterinary Sciences, James Cook University, Townsville, Queensland Australia; 3 Drowning Prevention Research Centre Canada, Toronto, Canada

**Keywords:** Drowning, Epidemiology, Coding systems, Public health, Mortality

## Abstract

**Background:**

Drowning is a leading and preventable cause of death that has suffered an attention deficit. Improving drowning data in countries would assist the understanding of the full extent and circumstances of drowning, to target interventions and evaluate their effectiveness. The World Health Organization identifies data collection as a key strategy underpinning effective interventions. This study compares unintentional fatal drowning data collection, management and comparison using the databases of Australia, Canada and New Zealand.

**Methods:**

Cases of fatal unintentional drowning between 1-January-2005 and 31–December-2014 were extracted. Cases were combined into a single dataset and univariate and chi square analysis (*p* < 0.01) were undertaken. Location and activity variables were mapped and combined. Variables consistently collected across the three countries were compared to the ILCOR Drowning Data Guideline. The authors also recommend variables for a minimum core dataset.

**Results:**

Of 55 total variables, 19 were consistent and 13 could be compared across the three databases. When mapped against the ILCOR Drowning Data Guideline, six variables were consistently collected by all countries, with five compared within this study. The authors recommend a minimum core dataset of 11 variables including age, sex, location, activity, date of incident, and alcohol and drug involvement).

There were 8176 drowning deaths (Australia 34.1%, Canada 55.9%, New Zealand 9.9%). All countries achieved reductions in crude drowning rates (Australia − 10.2%, Canada − 20.4%, New Zealand − 24.7%). Location and activity prior to drowning differed significantly across the three countries. Beaches (X^2^ = 1151.0;*p* < 0.001) and ocean/harbour locations (X^2^ = 300.5;p < 0.001) were common in Australia and New Zealand, while lakes/ponds (X^2^ = 826.5;*p* < 0.001) and bathtubs (X^2^ = 27.7;p < 0.001) were common drowning locations in Canada. Boating prior to drowning was common in Canada (X^2^ = 66.3;p < 0.001).

**Conclusions:**

The comparison of data across the three countries was complex. Work was required to merge categories within the 20% of variables collected that were comparable, thus reducing the fidelity of data available. Data sources, collection and coding varied by country, with the widest diversity seen in location and activity variables. This study highlights the need for universally agreed and consistently applied categories and definitions to allow for global comparisons and proposes a core minimum dataset.

## Background

Drowning is a leading and preventable cause of death that has suffered an attention deficit, due in part to a lack of quality data. The development of drowning prevention interventions requires data systems that provide detailed and nuanced information on the circumstances of drowning. The current estimate, acknowledged as an underreport of the true burden of drowning [1–5], is 372,000 fatal drownings globally per year [6]. Improving drowning data in countries has been identified as a key strategy by the World Health Organization (WHO) to better understand the full extent and circumstances of drowning, to target interventions and evaluate their effectiveness [7].

The use of International Classification of Diseases (ICD) codes to explore drowning is a common approach but provides a limited understanding of causal factors, impacting the development and reporting on the effectiveness of prevention strategies [8]. ICD-10 coding provides limited information on drowning location and provides no information on activity. Drowning deaths due to water transportation incidents, and as a result of flooding are also commonly excluded from global estimates [1].

The circumstances leading to drowning are complex. In high-income countries people interact with water primarily for recreation [9–13]. By contrast, drowning in low- and middle-income countries often occurs as a result of interactions with water due to daily life or occupational endeavours [14–16]. Prevention strategies, must therefore differ to suit the environment and the unique causal factors contributing to drowning risk in different contexts.

The comparison of data collation and database management methodologies has been used in other areas of research to work towards improving health [17–20]. Cross-country comparison of drowning data has also been conducted previously, with capture based only on ICD-10 codes [21]. Such comparisons are valuable to identify strengths, weaknesses and commonalities in data collation and coding, as well as enable sharing of successful interventions, across countries with similar drowning burden and risk factors. However the ability to compare relies on consistency of coding and/or transparency of definitions and frameworks used to collate and code data to allow for mapping. There currently exists no definitive coding framework for drowning data. The ILCOR Advisory Statement – 2015 Revised Utstein-Style Recommended Guidelines for Reporting of Data From Drowning-Related Resuscitation [22] (henceforth referred to as the ILCOR Drowning Data Guideline), through an established consensus process, proposes updated guidelines for reporting data from studies of resuscitation from drowning. These Guidelines can be considered a useful starting point for developing a minimum core dataset for drowning suitable for use in countries with and without death registry, although the Guidelines are used for improving clinical outcomes rather than prevention.

Death investigation is vital to understanding the epidemiology of drowning, due to the rich detail that can be derived from case documentation [23]. Australia, Canada and New Zealand, through the benefit of well-resourced death registry and coronial systems, have three of the best unintentional fatal drowning databases in the world. All are high-income, English speaking countries, with similar culture and population distribution. All have Indigenous populations at increased risk of drowning (Table 1).

**Table 1:** Country comparison

	Australia	Canada	New Zealand
Total Population	24,597,528 (June 2017)	36,708,083 (July 2017)	4,793,940 (June 2017)
Population aged 0–17 years	5,503,275 (22.4%)	7,058,050 (19.2%)	1,120,200 (23.4%)
Population aged 18–64 years	15,302,725 (62.2%)	23,454,489 (63.9%)	2,950,640 (61.5%)
Population aged 65 years and older	3,791,528 (15.4%)	6,195,544 (16.9%)	723,100 (15.1%)
Median age	38.7 years	42.2 years	37.9 years
Net migration (2017 est)	5.5 migrant(s)/1000 population	5.7 migrant(s)/1000 population	2.2 migrant(s)/1000 population
Ethnic makeup	English 25.9%, Australian 25.4%, Irish 7.5%, Scottish 6.4%, Italian 3.3%, German 3.2%, Chinese 3.1%, Indian 1.4%, Greek 1.4%, Dutch 1.2%, other 15.8% (includes Australian Aboriginal .5%), unspecified 5.4% #	Canadian 32.2%, English 19.8%, French 15.5%, Scottish 14.4%, Irish 13.8%, German 9.8%, Italian 4.5%, Chinese 4.5%, Indigenous Canadians 4.2%, other 50.9% *	European 71.2%, Maori 14.1%, Asian 11.3%, Pacific peoples 7.6%, Middle Eastern, Latin American, African 1.1%, other 1.6%, not stated or unidentified 5.4% ^
Total area (sq km)	7,741,220	9,984,670	268,838
Land area (sq km)	7,682,300	9,093,507	264,537
Water area (sq km)	58,920	891,163	4301
Coastline (kms)	25,760	202,080	15,134
Territorial sea (nautical miles)	12	12	12
Climate	Generally arid to semiarid: temperate in south and east; tropical in north	Varies from temperate in south to subarctic and arctic in north	Temperate with sharp regional contrasts
GDP per capita (2017 est)	$49,900	$48,100	$38,500

Population data: Australia (Australian Bureau of Statistics), Canada (Statistics Canada) and New Zealand (Statistics NZ). All other data from The CIA World Fact Book https://www.cia.gov/library/publications/the-world-factbook/

# Data represents self-identified ancestry, over a third of respondents reported two ancestries (2011 est) * Note percentages add up to more than 100% because respondents were able to identify more than one ethnic origin (2011 est) ^ Based on the 2013 census of the usually resided population; percentages add up to more than 100% because respondents were able to identify more than one ethnic group (2013 est)

This study aims to examine three of the most comprehensive fatal drowning databases in the world to: (1) describe data collection and coding; and (2) compare crude fatal drowning rates, demographics and risk factors.

## Methods

For the purposes of this study, only deaths where unintentional drowning was indicated as a primary or contributing cause of death were included. Excluded were all other water-related fatalities (i.e. spinal injury, hypothermia) where drowning was not a contributing factor. The process for the collection and collation of data in each of the three countries is discussed below.

### Australia

Data on all unintentional fatal drownings in Australia were sourced from the Royal Life Saving National Fatal Drowning Database [24] (the Australian database). All cases in the Australian database are extracted from the National Coronial Information System (NCIS), an online repository of all deaths investigated by a coroner. The NCIS provides rich detail identified using medico-legal investigation [23] made available in the form of up to four documents: a coroner’s report (or inquest report should an inquest occur), an autopsy report; a toxicology report and a police report. The data available on the NCIS varies (e.g. any combination of the four reports mentioned above) and relies on documentation being uploaded electronically onto the NCIS online system.

The Australian database also uses a triangulation method to source drowning data through year-round monitoring of media (print, broadcast, online) police reports, Child Death Review Team reports, social media and reports from lifesaving clubs [1].

Data collected (where available) includes: demographics (e.g. age, sex, residence, indigenous status, ethnicity etc), cause of death, circumstances (e.g. time of day, day of week, season, year, location, activity etc), autopsy and toxicology information (e.g. presence and type of pre-existing and contributory medical conditions, and drug and alcohol blood concentrations), as well as supplementary information on drowning related issues (e.g. lifejacket use, supervision, swimming skills). The Australian database includes all unintentional fatal drowning cases in Australia from 1-July-2002 and reports drowning on an annual financial year basis through the Royal Life Saving National Drowning Report [25] and a range of research outputs.

Data is transposed from the various sources into an IBMSPSS database for analysis. Data is correct as at 01-December-2017.

### Canada

Data on all unintentional fatal drowning incidents that occurred in Canada were obtained from the Drowning Prevention Research Centre (DPRC) database (the Canadian database). The Canadian database contains anonymized data related to all unintentional water-related fatalities that occur in Canada.

As part of an on-going water-related fatality surveillance project, trained local data collectors enter each of the provincial and territorial Coroner’s and Medical Examiner’s offices annually to conduct structured reviews of the files for all unintentional water-related deaths. Water-related death is defined as death from drowning [26], as well as all non-drowning death (e.g. hypothermia, trauma) involving water occurring while undertaking a range of activities. Although the Canadian database includes all unintentional water-related deaths, only those deaths where drowning was a primary or contributing cause of death were accessed for this study.

A structured questionnaire is used to collect data on demographics; cause of death; activity type and purpose of activity; and personal, equipment and environmental risk factors. The questionnaire was initially developed in 1991, and has been reviewed and revised approximately every 5years since then to ensure the reliability and comprehensiveness of the collected data. Data sources contained in the Coroner’s and Medical Examiner’s files vary by province and territory. Common documents found in the files and used to extract data include: Coroner’s or Medical Examiner’s investigation statement, death certificate, police report, hospital records, post-mortem/autopsy report, and toxicology report.

Data collectors fill out paper questionnaires that are reviewed by both a local and national project manager. When errors or inconsistencies are identified, the relevant file is re-opened and issues are addressed. Concerns of admissibility related to intentionality or cause of death are forwarded to a consultant epidemiologist and coroner for review and decision. Data entry of final validated cases occurs at the national level, and each case is subsequently reviewed for data entry errors.

The Canadian surveillance system utilises on-going review of media clippings to monitor current drowning trends. Water-related fatality cases identified in the media are entered into the database immediately after they occur. When cases from the Coroner’s and Medical Examiner’s files are entered into the database, they are automatically linked to the corresponding media file. This process assists with validation and verification of the cases and data.

Data is correct as at 17-January-2018.

### New Zealand

Water Safety New Zealand (WSNZ) are responsible for collating and maintaining details on all fatal drowning records in New Zealand (NZ). These records are kept in DrownBase™ which is the official database of WSNZ (the New Zealand database) [27]. The New Zealand database has been developed using Microsoft Access. The New Zealand database was developed in 1994 and contains records of all drowning deaths that have occurred in New Zealand’s waterways since 1 January 1980 and all water related hospitalisations requiring a stay in hospital of longer than 24 h since 2003.

Data is collated by WSNZ through partnerships with the New Zealand Police, the Coronial Services of the Ministry of Justice and the New Zealand Health Information Service (NZHIS). In order to maintain the integrity of the data, and to ensure WSNZ is accurately presented to the New Zealand public, a set protocol has been established. The protocol covers data collection and entry.

Fatal drowning data is initially collected from the New Zealand Police via *Drown reports and media reports. Follow up collection of data from Coroners’ reports, and Ministry of Health information assists in completing the data collection procedure.

The New Zealand database captures and records fatal drowning data across a range of fields. Where possible pre -existing lists are used, however some data is recorded by using a free text entry field, (eg. Family Name). All pre-existing lists are supported by comprehensive descriptors and metadata.

Preventable fatalities include recreational and non-recreational drowning deaths. They do not include those fatalities classified as ‘other’ (arising as a result of road or air vehicle accidents, homicide, suicide or of unknown origin) as these are not considered applicable to the prevention and rescue efforts of the water safety sector.

The word ‘intent’ could describe homicide or suicide. Unknown origin is a description used when we don’t how/why person ended up in the water.

Data is correct as at 29-November-2017.

### Country-comparisons

A subset of each country’s databases, (i.e. all fatal unintentional drowning cases for the 10 calendar year period 1-January-2005 to 31-December-2014), were extracted. The variables collected by each country were mapped (Table 2). Where all three countries collected the variable, it was copied across into a master IBMSPSS V20 [28] dataset. A new variable was added denoting the country in which the drowning occurred.

**Table 2:** Variables mapped across the three databases, Australia, Canada and New Zealand

Variable	Australia	Canada	New Zealand	Included in study (√)	Included in Utstein Guidelines (√) [23]	Recommended core variables (√)
Case ID	X	X	X		√ (Victim Identifier)	√
Data source			X			
Province, region, state/territory of drowning incident	X	X	X			
Incident year	X	X	X	√		
Date of incident (DD-MM-YYYY)	X	X	X	√	√ (Incident date and time of day)	√
Time of incident	X					
Primary Cause of Death	X	X				√
Contributory cause(s) of death	X	X				
Incident synopsis	X	X	X			
Incident type			X			
Inquest number			X			
Age in years	X	X	X		√ (Age -Birthdate)	√
Age group	X	X	X	√		
Sex	X	X	X	√	√ (Sex)	√
Ethnicity	X		X		√ (Race and ethnic categories)	√
First Nations Peoples	X	X	X	√		
Residential status	X	X	X	√		
Population Density (Urban/Rural)	X	X				
Location geographic	X	X	X			
Water Depth		X				
Distance from Safety		X				
Wave conditions		X				
Water current		X				
Ice conditions		X				
Water Temp Range		X				
Light conditions		X				
Aquatic location category	X	X	X	√		√
Activity prior to drowning	X	X	X	√		√
Purpose of Activity (Daily Living, Recreational/Non-recreational)		X	X			
Primary recreational activity		X				
Secondary recreational activity		X				
Daily Living Activity		X				
Occupational Activity		X				
Boating Causes		X				
Type of Boating Incident		X				
Watercraft Type	X	X				
Multiple Fatality Event – Yes/No/Unknown	X	X	X	√		
If yes, number of fatalities	X		X			
Lifejacket wear	X	X	X	√		
Medical Condition (pre-existing, chronic)	X	X	X	√	√ (Preexisting illness)	
Hypothermia			X			
Victim Alcohol Involvement	X	X	X	√		√
Blood Alcohol Content (BAC)	X	X				
Alcohol Involvement by Companions	X	X				
Victim Drug Involvement	X	X	X	√		√
Type of drug (legal, not legal)	X	X				
Type of drug (name)	X	X				
Victim swimming ability	X	X				
Resuscitation enacted – Yes/No/Unknown		X	X			√
Beach Patrolled			X			
Pool Fencing	X	X	X			
Supervision of children	X	X				
Commercial			X			
Occupation			X			
Purpose			X			

Location and activity codes as collected by each country were copied into the master dataset. They were then mapped across the three countries and similar categories were combined (Table 3) (Table 4). An amalgam of each country’s definitions were used to construct the definitions found in Tables 3 and 4.

**Table 3:** Location categories and definitions

Manuscript Category	Definition/Explanation	Australian label	Canadian label	New Zealand label
Bathtub	The victim was in a man-made tub or shower primarily used for personal bathing, generally emptied after use. Includes showers and indoor spa baths.	Bathtub	Bathtub	Bath
Beach	Sandy and rocky foreshore to coastal waters	Beach, Coastal Rocks		Surf Beach, Calm Water Beach, Rocky Foreshore
Lake/Pond	A body of water, of variable size, which is surrounded by land.	Lake/Dam/Lagoon, Pond	Lake or Pond	Pond, Lake
Ocean/Harbour	An open expanse of coastal water, characterised by tides, that is generally accessed via a jetty or watercraft. Excludes the sandy/rocky shore of a beach entry.	Ocean/Harbour	Ocean	0-1 km From Shore, 1-5 km From Shore, 5 + km From Shore, Marina, Harbour, River/Harbour Bar, Estuary
Other	Locations that do not fit into existing codes.	Other, Fishpond, Irrigation Channel, Drain	Other, Marsh/Bog/Swamp	Buckets, Drain, Other Waters, Domestic Location
River	A natural/fresh waterway fed by other bodies of water. Can vary in water flow, length, width and depth.	River/Creek/Stream	Flowing Water	Streams, Floods
Swimming Pool	A permanent or temporary excavation, structure or vessel that is solely intended, or principally used, for human aquatic activity. Includes portable, above and below ground pools in homes, hotels/motels and public areas. Includes outdoor spas.	Swimming Pool – Home, Swimming Pool – Public, Swimming Pool – Temporary Residence	Pool, Hot tub/Whirlpool	Thermal Pools, Spa Pools, School Pools, Public Pools, Portable Pools, Hotel/Motel Pools, Home Pools
Unknown	Location of drowning incident is not known	Unknown	Unknown	Unknown

**Table 4:** Activity categories and definitions

Manuscript Category	Definition/Explanation	Australian label	Canadian label	New Zealand label
Aquatic activity	The victim was in the water and intended to be there.	Swimming and Recreating, Diving, Fishing, Jumped In	Aquatic activity	Free diving, Snorkelling, Scuba Diving, Diving/Jumping, Net fishing, Commercial fishing, Angling, Shellfishing, Swimming, Boogie boarding, board riding, Tubing, canyoning,
Bathing	Submerging/immersing the body in water for the purposes of relaxation or cleaning. Generally not vigorous activity.	Bathing	Bathing	Bathing
Boating	Using a powered or unpowered vessel for the purposes of recreation or transportation.	Boating, Watercraft	Boating	Windsurfing, Under 4 m, Trailer Sailor, Sailing Dinghy, Rowing craft/Dinghy, Rafting, Over 4 m, Offshore sailing, Kayaking, Jet skis, Jet boat, Fixed keel boat, Canoeing,
Non-aquatic activity	The victim did not intend to be in the water at the time of the incident, was near or on the water or ice.	Fall, Swept In, Rock Fishing, Swept away, Rescue	Non-aquatic activity	Accidental immersion, Flood/civil emergency, Rescuing others
Non-aquatic transport	Operating (or a passenger in) any kind of vehicle not intended for aquatic activity, such as motor vehicles, snowmobiles, aircraft etc.	Non-aquatic transport	Land, ice or air transportation	
Other	Unable to be classified in other categories.	Other		Other recreation
Unknown	Where activity immediately prior to drowning is not known.	Unknown	Unknown	Unknown

Alcohol refers to where alcohol was present (yes, no, unknown) rather than blood alcohol concentrations (BAC). Drugs (yes, no, unknown) refers to both legal (e.g. prescription) and illicit (illegal) drug consumption. International tourists are those people who were known to reside in a different country from the country where they drowned. A multiple fatality event (MFE) refers to a single drowning incident where more than one person drowned. Lifejacket wear was only collected and analysed for boating drowning incidents (*n* = 2039). Indigenous peoples is used as a catch-all term to refer to Maori, Australian First Nations Peoples and Canada’s Indigenous populations.

Variables collected were compared to those recommended in the ILCOR Drowning Data Guideline [22]. The authors also make recommendations for those variables to be included in a minimum core dataset for countries compiling drowning databases with a view to enabling better comparison of drowning data across countries and contributing to drowning prevention.

### Statistical analysis

Nineteen of the 55 variables made available were consistently reported by all three countries and 13 were able to be compared in this study. There were six variables consistently collected, but not compared within this study: region/location/state or territory of drowning incident within a country, incident synopsis, age in years, location geographic and pool fencing. Region of drowning incident, incident synopsis and location geographic all varied and were not able to be compared between countries. Age in years was used to calculate age group for ease of analysis. Pool fencing was not included within the scope of the study as it is a drowning prevention strategy most suitable for children under five and as such, was not comparable across all age groups.

Univariate and chi square analysis was undertaken. Statistical significance was deemed *p* < 0.001. Non-parametric testing was also conducted using the proportional basis of the population as the assumed outcome numbers. Crude annual drowning rates per 100,000 population and 10 year averages were calculated for each country using population data sourced from national statistics organisations [29–31]. Drownings of international tourists (without a residential address) were removed from the numerator when calculating rates as they were not represented in the population estimates. Those cases where residential status was unknown were retained.

## Results

Of the 55 variables made available for this study, 19 were consistent across all three databases. Consistently collected variables included date of incident, age group, sex, residential status, category of aquatic location, activity prior to drowning and if the drowning occurred during a MFE, among others (Table 2). When mapped against the ILCOR Drowning Data Guideline [22], 6 variables were consistently collected by all countries, with five being able to be compared within this study. The five compared within the study were date of incident (referred to in ILCOR Data Guideline as incident date and time of day); age (age – birthdate); sex (sex); first nation’s peoples (race and ethnic categories); and medical condition (pre-existing illness). The variable of case ID (victim identifier) was consistently collected but not able to be compared (Table 2).

The authors recommend a minimum core dataset of 11 variables. The recommended variables are: Case ID; date of incident; primary cause of death; age in years (used to code to age group); sex; ethnicity; aquatic location category; activity prior to drowning; victim alcohol involvement; victim drug involvement; and resuscitation enacted – yes/no/unknown (Table 2).

There were 51 categories collected across the three countries for location of drowning (ranging from nine to 27). (Table 3) Activity being undertaken immediately prior to drowning was classified into 53 categories across the three countries. The number of activity categories ranged from six in Canada to 32 in New Zealand (Table 4).

A total of 8176 cases of unintentional fatal drowning occurred in the three countries (Australia 34.1%, Canada 55.9%, New Zealand 9.9%). Crude fatal drowning rates per 100,000 population have been as low as 1.06 in Australia in 2014 and as high as 2.21 in New Zealand in 2008. When comparing the relative change between the first and the last year’s drowning rates for each nation, all three have achieved reductions, with the highest reduction seen in New Zealand (− 24.7%) (Table 5).

**Table 5:** Resident crude drowning rates per 100,000 population by individual year, 10 year average and % change, Australia, Canada and New Zealand

	Australia	Canada	New Zealand
2005	1.18	1.42	1.86
2006	1.27	1.46	1.65
2007	1.31	1.38	1.97
2008	1.11	1.38	2.21
2009	1.31	1.29	1.98
2010	1.21	1.31	1.43
2011	1.15	1.22	2.14
2012	1.12	1.37	1.68
2013	1.16	1.25	1.58
2014	1.06	1.13	1.42
10 year average	1.19	1.32	1.79
% change (2014 vs 2005)	−10.2%	−20.4%	−24.7%

Note: Drowning deaths of international tourists have been removed for the purposes of rate calculations

The proportion of child drowning ranged from 12.1% in Canada to 17.6% in Australia (X^2^ = 46.4;*p* < 0.001). There was no difference in the proportion of people aged over 50 years drowning by country (Table 6).

**Table 6:** Demographics, activity and location of fatal unintentional drowning, Australia, Canada and New Zealand

	Australia		Canada		New Zealand		X^2^ (*p* value)
	N	%	N	%	N	%	
Total	2792	34.1	4572	55.9	812	9.9	118.349 (p < 0.001)
Sex (*N* = 8176)							
Male	2182	78.2	3715	81.3	669	82.4	13.027 (*p* = 0.001)
Female	610	21.8	857	18.7	143	17.6	
Age group (*n* = 8174)							
Children (0–17 years)	492	17.6	555	12.1	138	17.0	46.431 (p < 0.001)
Adults (18–64 years)	1254	44.9	2220	48.6	376	46.3	9.622 (*p* = 0.008)
Older people (65 years and older)	1046	37.5	1795	39.3	298	36.7	3.470 (*p* = 0.176)
International tourists (N = 8176)							
Yes	132	4.7	122	2.7	40	4.9	28.917 (p < 0.001)
No	2606	93.3	4426	96.8	706	86.9	
Unknown	46	1.6	24	0.5	66	8.1	–
First Nations Peoples (N = 8176)							
Yes	156	5.6	503	11.0	194	23.9	195.631 (p < 0.001)
No	2232	79.9	4033	88.2	597	73.5	
Unknown	382	13.7	36	0.8	21	2.6	–
Aquatic location of drowning incident (N = 8176)							
Bathtub	203	7.3	444	9.7	40	4.9	27.655 (p < 0.001)
Beach	636	22.8	0	0.0	181	22.3	1150.963 (p < 0.001)
Lake/Pond	251	9.0	1665	36.4	72	8.9	826.485 (p < 0.001)
Ocean/Harbour	435	15.6	421	9.2	254	31.3	300.481 (p < 0.001)
Other	99	3.5	334	7.3	23	2.8	59.527 (p < 0.001)
River	756	27.1	1282	28.0	183	22.5	10.643 (*p* = 0.005)
Swimming Pool	412	14.8	423	9.3	59	7.3	66.262 (p < 0.001)
Unknown	0	0.0	3	0.1	0	0.0	–
Activity immediately prior to drowning (N = 8176)							
Aquatic activity	793	28.4	1171	25.6	355	43.7	72.757 (p < 0.001)
Bathing	201	7.2	437	9.6	0	0.0	91.064 (p < 0.001)
Boating	479	17.2	1098	24.0	167	20.6	37.105 (p < 0.001)
Non-aquatic activity	839	30.1	906	19.8	287	35.3	165.660 (p < 0.001)
Non-aquatic transport	183	6.6	740	16.2	0	0.0	266.668 (p < 0.001)
Oher	20	0.7	0	0.0	3	0.4	96.587 (p < 0.001)
Unknown	277	9.9	220	4.8	0	0.0	–

New Zealand (4.9%) and Australia (4.7%) recorded a significant proportion of fatal drownings among international tourists (X^2^ = 28.9;*p* < 0.001). Twenty-four percent (23.9%) of drowning deaths in New Zealand were Indigenous peoples (X^2^ = 195.6;*p* < 0.001) (Table 6).

There were differences in locations of drowning in each country. Rivers were the leading location for drowning in Australia (27.1%), lakes/ponds in Canada (36.4%) and ocean/harbour locations in New Zealand (31.3%). Australia was the only country to record a statistically significant drowning burden in swimming pools (X^2^ = 66.3;*p* < 0.001) (Table 6).

Activity prior to drowning also varied, with aquatic activity common in Australia (28.4%) and New Zealand (43.7%) (X^2^ = 72.8;p < 0.001) and bathing (9.6%; X^2^ = 91.1;*p* < 0.001) and boating-related incidents (24.0%; X^2^ = 37.1;p < 0.001) common in Canada (Table 6).

Alcohol was a common risk factor with known involvement in 36.0% of Canadian drownings and 25.8% of Australian drowning. Drugs were most commonly involved in Australia (27.0%; X^2^ = 350.5;p < 0.001) and Canadian drowning fatalities (24.4%). Pre-existing medical conditions were present in a similar proportion of cases in Australia (37.2%) (X^2^ = 304.5;p < 0.001) and Canada (37.1%) (Table 7).

**Table 7:** Alcohol, drugs, medical conditions, lifejacket wear, multiple fatality event, Australia, Canada and New Zealand

	Australia		Canada		New Zealand		X^2^ (p value)
	N	%	N	%	N	%	
Total	2792	34.1	4572	55.9	812	9.9	118.349 (p < 0.001)
Alcohol involvement							
Yes	719	25.8	1647	69.0	133	16.4	106.674 (p < 0.001)
No	1203	43.1	2465	21.0	551	67.9	
Unknown	729	26.1	460	10.1	128	15.8	–
Drug involvement							
Yes	755	27.0	1117	24.4	18	2.2	350.481 (p < 0.001)
No	1060	38.0	2397	52.4	666	82.0	
Unknown	729	26.1	1058	23.1	128	15.8	–
Medical condition (pre-existing, chronic)							
Yes	1039	37.2	1698	37.1	153	18.8	304.496 (p < 0.001)
No	739	26.5	2694	58.9	492	60.6	
Unknown	853	30.6	180	3.9	167	20.6	–
Lifejacket wear (n = 2039) #							
Yes	42	9.4	210	15.8	51	19.1	2.099 (*p* = 0.350)
No	155	34.8	894	67.4	171	64.0	
Unknown	249	55.8	222	16.7	45	16.9	–
Multiple Fatality Event							
Yes	254	9.1	717	15.7	90	11.1	67.703 (p < 0.001)
No	2528	90.5	3854	84.3	701	86.3	
Unknown	10	0.4	1	0.0	21	2.6	–

#Boating only, excludes lifejackets worn in association with rock fishing and other activities where lifejackets are not applicable

Canada recorded the highest proportion of drowning deaths where lifejackets were not worn (67.4%). Sixteen percent (15.7%) of Canada’s drowning fatalities during the study period occurred as a result of a MFE (X^2^ = 67.7;p < 0.001) (Table 7).

## Discussion

Drowning prevention is a “wicked problem” [32] with a range of risk factors requiring targeted prevention strategies [33, 34]. Robust high-quality data underpins evidence for effective policy, interventions and education [34]. Currently there is a move across high-income countries to develop sophisticated drowning data systems [1, 11]. This study compares the drowning data collection of three countries who have similar language, culture, aquatic engagement and drowning prevention. Better data collection systems should enable the creation of targeted and effective drowning prevention interventions. It is likely that, as systems grow, more variables will be collected. However, reliability and validity of these variables will need to be tested, and then likely reduced to a core set of important indicators, such as those suggested in the ILCOR Drowning Data Guideline [22], with local variation enabled to address specific challenges.

There currently exists no definitive guide to drowning data collection and coding variables (including sub-categories). The ILCOR Drowning Data Guideline [22], although focusing on reporting data associated with drowning-related resuscitation, represents a useful first step. Six variables collected by countries within this study directly map to the ILCOR recommendation (five of which were able to be compared), however for those focused on drowning prevention, key variables required for the development of drowning prevention strategies are not discussed. For those countries focused on collecting and collating drowning data with a view to prevention, information on drowning causal factors is vital, including detail on aquatic location and activity being undertaken immediately prior to drowning. As a result, the authors of this study propose a core minimum dataset of 11 variables including age, sex, location, activity, date of incident, primary cause of death and drug and alcohol involvement). Such variables are included based on a philosophy of data collection for drowning prevention and quality and availability of data. As countries become more sophisticated and drowning data collection matures, further variables can be added, however this core dataset should be maintained. Across the study period, 8176 cases of fatal, unintentional drowning were captured, with all three countries achieving reductions in crude drowning rates of at least 10%. Detailed comparison across the three countries was challenging due to differing definitions, coding and data collected. It appears that the risk factors for drowning are similar across the countries with males (80.3%), drugs (23.1%), alcohol (30.6%) and pre-existing medical conditions (35.3%) contributing to the drowning burden. There were however, local variations between the datasets. For example Australia and New Zealand saw significant (*p* < 0.001) numbers in ocean/harbour and beach locations, whereas in Canada, drownings were more likely (p < 0.001) to occur in lakes/ponds and bathtubs.

For effective cross-country comparative studies, availability of quality data and comparable coding frameworks will be required, including coding hierarchy especially for cases where interpretation and therefore coding, could vary. Aside from basic drowning descriptors such as location and activity, all three countries collected rich information on risk and causal factors [17, 35] including data related to Indigenous peoples and lifejacket use. This suggests that all three countries identify Indigenous peoples as being at increased risk and lifejackets as an effective prevention strategy. With increased understanding of drowning risk factors, there will be an international need for minimum datasets and consistent coding.

Data quality poses a challenge for all undertaking epidemiological research. To help improve drowning data quality, there will be a need for engagement with the medico-legal process, including medical examiner, police and others involved in investigating drowning deaths. At a population level, ensuring the routine testing for, and collection of, data on presence of alcohol, drugs and pre-existing medical conditions will be vital for evidence-based prevention efforts.

The comparison of drowning data between countries allows for identification of similarities in drowning risk and therefore effective prevention, as well as informing potential improvements in data collection and coding.

Calculation of country-specific drowning rates would exclude non-residents. However, there is increasing concern for, and a need to develop an understanding of, those who drown outside their country of residence. There are two broad groupings of people: (1) tourists; and (2) refugees, migrants and people without a fixed address. In Canada visitor status is not routinely collected and had to be derived for this study. Though often small numbers (international tourists account for 4% of drowning deaths in Australia [36]) they are a unique subset requiring different prevention strategies.

With increasing focus on drowning among refugees, migrants and stateless peoples [37–41], there is a unique challenge to calculate drowning rates worldwide. In Canada, during the study period three refugees drowned during a migration attempt and two illegal immigrants drowned. Drowning among non-residents is a challenge for prevention.

Collating and comparing data on sex and age group is reasonably straightforward whereas activity and location is more challenging. Within the three databases the number of categories within a variable differed, thus fidelity was lost, when merged. Detailed activity and location coding (i.e. greater than currently available in ICD-10 and more like what is proposed for ICD-11) will be critical for future international comparative studies. Alternative coding frameworks, such as that proposed by the International Classification of External Causes of Injury (ICECI) Coordination and Maintenance Group [42], provides greater detail and therefore more detailed evidence to support prevention efforts.

## Strengths and limitations

A strength is the drowning data represents all cases of unintentional fatal drowning, beyond the narrow inclusion criteria based only on primary cause of death with an ICD code of W65–74, which has been found to underreport drowning [1, 3, 43]. This however, means that outside of the three countries included in this study, data is not comparable unless the same inclusion criteria is applied. The data is longitudinal, yet retrospective in nature and is drawn from the coronial system, allowing causal and risk factor analysis to be conducted. Data in this study is based on cause of death and not ICD-10 coding, and may therefore differ from official cause of death statistics.

Fatal drowning data from all three countries is coronial data and details for cases which are open (i.e. under investigation) may change. Only those variables where comparison was possible have been included in the study. Assumptions have been made when mapping location and activity variables. Those drowning victims with unknown residential status or those on work or student visas have been retained for the purposes of rate calculations.

## Conclusion

Drowning is a global public health threat, impacting both high income and low and middle income countries. A total of 8176 drowning deaths were recorded across the study period, with all countries achieving at least a 10% reduction in country-level crude drowning rates across the 10 year period of the study. Among the data coding methodologies used by the three countries, there were 55 variables available for analysis of which, 19 were consistently collected and 13 were comparable. To compare the variables of location and activity, there was a need to merge categories, thus reducing the fidelity of data available. Future studies involving more countries will require work to enable comparisons to occur. The authors have identified 11 variables that would form a core minimum dataset, however there is a need for the development and validation of category definitions for location and activity variables that are consistently applied to allow for global comparison.

## Data Availability

Coronial data used in this study is not publicly available due to its sensitive nature. Therefore, the dataset supporting the findings in this study cannot be shared unless the person has obtained ethical approval due to the terms of the ethics agreement under which each of the country’s data was approved (please see ethics approval and consent to participate section). Applications for access to the data can be made to apeden@rlssa.org.au (Australia), experts@drowningresearch.ca (Canada) and wsnz@watersafety.org.nz (New Zealand). The Canadian questionnaire is also not publicly available, but access can be requested by email: experts@drowningresearch.ca.

## Abbreviations

BAC: Blood Alcohol Concentration
DPRC: Drowning Prevention Research Centre
IBMSPSS: International Business Machines Statistical Package for the Social Sciences
ICD: International Classification of Diseases
ICECI: International Classification of External Causes of Injury
MFE: Multiple Fatality Event
NCIS: National Coronial Information System
NZHIS: New Zealand Health Information Service
WHO: World Health Organization
WSNZ: Water Safety New Zealand
